# Facial Assessment and Cosmetic Enhancement Quality of Life Questionnaire (FACE-Q) Arabic Validation

**DOI:** 10.7759/cureus.51135

**Published:** 2023-12-26

**Authors:** Mohammd Alsanea, Yazeed J Alshaalan, Mohammad Alkraidees, Rana Alramyan, Shaikh K Waheed

**Affiliations:** 1 Otolaryngology - Head and Neck Surgery, King Abdulaziz Medical City, Riyadh, SAU; 2 Otolaryngology - Head and Neck Surgery, King Abdullah International Medical Research Center, Riyadh, SAU; 3 Otolaryngology, Ministry of National Guard Health Affairs, Riyadh, SAU; 4 Otolaryngology - Head and Neck Surgery, King Saud Bin Abdulaziz University for Health Sciences, Riyadh, SAU; 5 General Practice, King Saud Bin Abdulaziz University for Health Sciences, Riyadh, SAU; 6 General Practice, King Abdullah International Medical Research Center, Riyadh, SAU; 7 Otolaryngology, King Saud Bin Abdulaziz University for Health Sciences, Riyadh, SAU; 8 Otolaryngology, King Abdullah International Medical Research Center, Riyadh, SAU; 9 Otolaryngology, King Saud Bin Abdulaziz University for Health Sciences College of Medicine, Riyadh, SAU; 10 Otolaryngology - Head and Neck Surgery, Ministry of National Guard Health Affairs, Riyadh, SAU

**Keywords:** septoplasty, questionnaire development and validation, aesthetic facial surgery, rhinoplasty, face-q

## Abstract

Background

Rhinoplasty, a common cosmetic surgery, improves aesthetic appearance and nasal function. Outcomes are typically measured by patient satisfaction and quality of life impact. However, a gap exists in validated non-English assessment tools, especially in Arabic, which hinders accurately capturing patient experiences in Arabic-speaking populations. To fill this gap, this study aims to translate and validate the "Rhinoplasty" module of the Facial Assessment and Cosmetic Enhancement Quality of Life Questionnaire (FACE-Q) self-questionnaire into Arabic.

Methods

A cross-sectional study was conducted at the Otorhinolaryngology Department of the National Guard Health Affairs in Riyadh, Saudi Arabia. Adults who underwent rhinoplasty from 2017 to 2021 were included if they had at least one year of follow-up and were contactable. A sample size of 109 was determined, and the participants were selected using probability cluster sampling. A 33-item FACE-Q scale was administered via telephone, with scores converted to a 0-100 scale for analysis. Translation involved a two-way process with independent translations and back-translations, followed by review and pilot testing.

Results

The study included 137 participants (mean age, 32.5 years; 53 men, 84 women), predominantly electing cosmetic surgery, with an average of four years post-surgery. The internal consistency of the scales varied, with the self-acceptance/love scale showing acceptable reliability (Cronbach’s alpha = 0.73) and other scales suggesting item redundancy (Cronbach’s alpha for aesthetic scales > 0.94). The self-rated complications scale did not meet the acceptability threshold, indicating a need for scale revision.

Conclusions

The Arabic translation of the FACE-Q Rhinoplasty module shows potential as a reliable tool for evaluating patient satisfaction and quality of life after rhinoplasty in Arabic-speaking patients. Further refinement is necessary to address item redundancy and enhance cultural specificity. This work underscores the importance of culturally sensitive tools for global applicability in cosmetic surgery outcomes research.

## Introduction

Assessing outcomes post-rhinoplasty is a critical element of cosmetic surgery that gauges patient satisfaction and the effect of surgical interventions on the quality of life [1,2]. The challenge lies in the scarcity of aesthetic and quality-of-life assessment tools in non-English languages, notably Arabic [3-6]. Adapting these tools to various languages, including Arabic, is vital for their validity and global applicability, enabling the capture of diverse patient experiences [7-9].

Rhinoplasty, the surgical reshaping of the nose, is a prevalent cosmetic surgery that enhances both aesthetic appeal and nasal function [10,11]. The procedure modifies the nose’s size, shape, and structure to meet the patient’s expectations [10]. Besides aesthetic improvement, rhinoplasty can rectify functional issues, such as breathing impairments or nasal blockages [12,13]. A thorough evaluation of rhinoplasty outcomes encompasses patient-reported outcomes and satisfaction levels [1,14].

Undergoing a successful rhinoplasty can help patients improving their self-confidence. The Facial Assessment and Cosmetic Enhancement Quality of Life Questionnaire (FACE-Q) is a validated self-completed instrument recognized for evaluating patient satisfaction and the impact of facial cosmetic surgeries [15]. Its "Rhinoplasty" module, created by Klassen et al., specifically assesses patient contentment with their nasal pyramid and nasal wings [16]. This module is instrumental for discerning the success of rhinoplasty and understanding its influence on patients’ lives.

This study aims to translate and validate the "Rhinoplasty" module of the FACE-Q self-questionnaire into Arabic, catering to Arabic-speaking patients. We intend to establish a standardized, culturally adapted tool to assess patient-reported outcomes and satisfaction post-rhinoplasty in the Arabic-speaking demographic. Providing the Arabic version of the FACE-Q will improve the evaluation of rhinoplasty outcomes, offering healthcare professionals and researchers insights into the procedure’s success in this population.

## Materials and methods

We conducted the research at the Otorhinolaryngology Department, Facioplasty Clinic, National Guard Health Affairs, Riyadh, Saudi Arabia. Eligible participants were adults who underwent rhinoplasty between 2017 and 2021 at the institution and had a verifiable contact number. We excluded patients with less than one year of follow-up or those unreachable by telephone.

We employed a cross-sectional design to collect data. Using the Raosoft online calculator (Raosoft, Inc., Seattle, WA), we estimated a sample size of 109 based on a 5% margin of error, a 95% confidence level, and a target population of 150 patients over four years. We used probability cluster sampling to select participants. Patients who had rhinoplasty and were reachable by phone were invited to participate. Those who consented were included.

Data collection involved a questionnaire administered via phone by co-investigators, including a demographic section for age and gender. We used the 33-item Face-Q scale, rated on a four-point scale, to assess outcome satisfaction. We converted scores to a 0-100 scale to derive an overall satisfaction score.

We manually entered data into IBM SPSS Statistics for Windows, version 22.0. (released 2013, Armonk, NY: IBM Corp.) for analysis. We presented categorical variables as frequencies (percentages) and numerical data as mean ± standard deviation (SD). We used the chi-squared test for categorical variable associations and the student’s t-test for comparing scores with variables like gender. A p-value of <0.05 indicated statistical significance.

For the questionnaire’s Arabic translation, a rhinoplasty specialist and a professional translator, both Arabic native speakers fluent in English, independently translated the text. We then back-translated this initial Arabic version into English by a native English speaker and compared it with the original FACE-Q. After resolving discrepancies through iterations and development team input, a panel of healthcare professionals reviewed the comprehensibility, leading to further refinement. We tested the revised Arabic version on 25 patients, with practitioner feedback guiding further modifications. The finalized version underwent review and validation by a panel of surgeons, with minor adjustments finalizing it for use in subsequent studies and assessments.

## Results

The dataset included 137 participants with an average age of 32.5 years (SD = 7.60). Among them, there were 53 men with a mean age of 31.9 years (SD = 6.73) and 84 women with a mean age of 32.9 years (SD = 8.12). We gathered data on the motivations for undergoing rhinoplasty. Cosmetic elective procedures emerged as the predominant reason, constituting 22% of the total sample. Injuries were the second most cited cause, making up 18% of the cases, while 14% involved patients seeking to address structural deformities through reconstruction and restoration. Septorhinoplasty and the correction of nasal deviations were each responsible for 7% of the interventions. The mean time since the surgeries was 4.0 years (SD = 1.9), with the most common interval being five years (Table 1). 

**Table 1:** Time since surgery

Time since surgery (years)	Patients (n)
Within 1 year	16
Within 2 years	20
Within 3 years	23
Within 4 years	22
5	25
> 5	31

The evaluation of the scales revealed that the self-acceptance/love scale, consisting of 18 items, showed acceptable internal consistency with a Cronbach’s alpha of 0.73. Elimination of certain items could potentially raise this value to 0.75 or even 0.8. The aesthetic scales for the face and the nose and nostrils, comprising 10 and four items, respectively, showed high alpha values of 0.95 and 0.94. These high values suggest the need to review the scales for item redundancy. The self-rated complication scale, including four items, did not meet the threshold of acceptability, presenting a Cronbach’s alpha of 0.59. The satisfaction with the outcome scale, which contained six items, exhibited a very high Cronbach’s alpha of 0.96, again indicating possible item redundancy. These outcomes underscore the necessity of examining the scales’ purposes, measurement objectives, and the interpretation of scores for their reliability. Table 2 summarizes these statistical findings.

**Table 2:** Cronbach's alpha values for the FACE-Q rhinoplasty module scales in an Arabic-speaking sample Abbreviation: FACE-Q, Facial Assessment and Cosmetic Enhancement Quality of Life Questionnaire

Name of the scale	Number of questions	Cronbach's alpha (internal consistency) of the questions	Notes
Self-acceptance/love scale	18	.73	Acceptable. The internal consistency would increase to .75 if any of the last eight questions are removed. Removing two of the last eight questions will increase it to .8
Aesthetics (face)	10	.95	Acceptable. However, the alpha is too high. A recommended upper value of the alpha is .90; a higher alpha suggests that the questions are repeated.
Aesthetics (nose)	10	.94	Acceptable. However, the alpha is too high. A recommended upper value of the alpha is .90; a higher alpha suggests that the questions are repeated.
Aesthetics (nostrils)	5	.94	Acceptable. However, the alpha is too high. A recommended upper value of the alpha is .90; a higher alpha suggests that the questions are repeated.
Self-rated complications (alterations)	4	.59	Not acceptable. However, these four questions are better to be statistically treated as individual items as each question entails a separate fact, and the items combined do not reflect a combined scale.
Satisfaction with the outcome	6	.96	Acceptable. However, the alpha is too high. A recommended upper value of the alpha is .90; a higher alpha suggests that the questions are repeated.

## Discussion

This study aimed to assess the outcomes of rhinoplasty by measuring patient satisfaction with their facial appearance, quality of life post-surgery, and any adverse events associated with the procedure. Evaluating Arabic patients with an Arabic questioner will help to understand the impact of the surgery. We used the Arabic version of the FACE-Q scale to assess patient satisfaction, scrutinizing its psychometric properties, reliability, and validity. The internal consistency of the various scales ranged from acceptable to high, with Cronbach’s alpha values between 0.73 and 0.96. However, the high alpha values in some scales suggested item overlap, which calls for additional analysis and refinement. It is crucial to align these results with those from other studies in different languages to affirm their consistency and general applicability.

Cultural considerations may affect responses to the FACE-Q items [17-19]. The translation process incorporated measures to ensure cultural relevance and translation accuracy. Native Arabic speakers, including a rhinoplasty specialist and a translator, executed the initial translation. Back-translation, iterative revisions, and feedback from a panel of healthcare professionals and 25 patients were integral to the questionnaire’s refinement. This approach was designed to honor cultural nuances and verify the scale’s suitability for Arabic-speaking patients [17].

The study’s implications extend to research and clinical practices. The Arabic FACE-Q has shown to be a valid and reliable tool for assessing patient-reported outcomes in facial aesthetics. This tool can aid in research evaluating cosmetic procedure effectiveness in Arabic-speaking individuals. It also serves as a critical instrument for healthcare providers to measure patient satisfaction and quality of life, thus informing personalized treatment strategies. However, the discovery of possible item redundancy necessitates ongoing research to fine-tune the FACE-Q for this demographic.

This study had several important limitations. It was conducted within the confines of a singular clinical setting, which may constrain the extrapolation of the findings to a wider context. Accordingly, it is recommended that subsequent research endeavors extend their scope to incorporate a broader and more varied array of participants from multiple clinical environments. In addition, the study’s reliance on self-reported data, in the absence of corroborating clinical evaluations of surgical success, may limit the objectivity of the outcome measures. The influence of patients’ health literacy on their comprehension and responses to the questionnaire was not assessed and remains unexplored in this study.

Furthermore, there was no standardization for surgical techniques or consideration of the surgeons’ expertise, both of which can significantly sway the results. Another limitation is the data collection method through telephone interviews, which can introduce the possibility of response bias, with participants possibly inclined to provide answers that they perceive as socially acceptable. In addition, the recall bias cannot be dismissed, given the four-year average interval since surgery, which could potentially compromise the recollection accuracy of the participants. In addition, while meticulous efforts were made to tailor the Arabic version of the FACE-Q culturally, future studies must engage in more thorough validation to ascertain its cross-cultural applicability. Finally, longitudinal research is essential to glean a more robust understanding of the long-term effects of rhinoplasty on patient-reported outcomes within Arabic-speaking populations.

## Conclusions

This investigation enhances the understanding of patient-reported outcomes post-rhinoplasty among Arabic-speaking populations. The Arabic version of the FACE-Q demonstrates promise as a reliable and valid instrument for gauging patient satisfaction and quality of life. Further refinement and validation are required to address potential item redundancy and cultural specificity. The results underscore the significance of continued research and creating culturally sensitive assessment tools to optimize patient care and outcomes in rhinoplasty.
